# Infodemiology of Alcohol Use in Hong Kong Mentioned on Blogs: Infoveillance Study

**DOI:** 10.2196/jmir.2180

**Published:** 2013-09-02

**Authors:** KL Chan, SY Ho, TH Lam

**Affiliations:** ^1^School of Public Health, The University of Hong KongHong KongChina (Hong Kong)

**Keywords:** alcohol drinking, blogging, blog search, Chinese, Hong Kong, infodemiology, infoveillance, Internet

## Abstract

**Background:**

In 2007 and 2008, the beer and wine tax in Hong Kong was halved and then abolished, resulting in an increase of alcohol consumption. The prevalence of the Internet and a high blogging rate by adolescents and adults present a unique opportunity to study drinking patterns by infodemiology.

**Objective:**

To assess and explain the online use of alcohol-related Chinese keywords and to validate blog searching as an infoveillance method for surveying changes in drinking patterns (eg, alcohol type) in Hong Kong people (represented by bloggers on a Hong Kong–based Chinese blogging site) in 2005-2010.

**Methods:**

Blog searching was done using a blog search engine, Google Blog Search, in the archives of a Hong Kong–based blog service provider, MySinaBlog from 2005-2010. Three groups of Chinese keywords, each representing a specific alcohol-related concept, were used: (1) “alcohol” (ie, the control concept), (2) “beer or wine”, and (3) “spirit”. The resulting blog posts were analyzed quantitatively using infodemiological metrics and correlation coefficients, and qualitatively by manual effort. The infodemiological metrics were (1) apparent prevalence, (2) actual prevalence, (3) prevalence rate, and (4) prevalence ratio. Pearson and Spearman correlations were calculated for prevalence rates and ratios with respect to per capita alcohol consumption. Manual analysis focused on (1) blog author characteristics (ie, authorship, sex, and age), and (2) blog content (ie, frequency of keywords, description of a discrete episode of alcohol drinking, drinking amount, and genres).

**Results:**

The online use of alcohol-related concepts increased noticeably for “alcohol” in 2008 and “spirit” in 2008-2009 but declined for “beer or wine” over the years. Correlation between infodemiological and epidemiological data was only significant for the “alcohol” prevalence rate. Most blogs were managed by single authors. Their sex distribution was even, and the majority were aged 18 and above. Not all Chinese keywords were found. Many of the blog posts did not describe a discrete episode of alcohol drinking and were classified as personal diary, opinion, or emotional outlets. The rest lacked information on drinking amount, which hindered assessment of binge drinking.

**Conclusions:**

The prevalence of alcohol-related Chinese keywords online was attributed to many different factors, including spam, and hence not a specific reflection of local drinking patterns. Correlation between infodemiological data (represented by prevalence rates and ratios of alcohol-related concepts) and epidemiological data (represented by per capita alcohol consumption) was poor. Many blog posts were affective rather than informative in nature. Semantic analysis of blog content was recommended given enough expertise and resources.

## Introduction

### Alcohol Use and Tax Policies in Hong Kong

Although often overlooked, alcohol is a human carcinogen [1]. It causes 2.5 million deaths each year worldwide [2], and the United Nations has identified its harmful use as one of the four most important risk factors for noncommunicable diseases [3]. In Hong Kong, although alcohol consumption is still low, it is not uncommon. Alcohol is easily accessible by the general population due to a lack of regulation of minimum age for off-premises sales (with “premises” defined as restaurants and bars granted with a liquor license) [4,5]. Local studies have shown that almost one-third of adults and one-fourth of secondary school students drink alcohol [6]. The adverse health effects are daunting. According to the data released by the Department of Health in 2006-10, there was an annual average of more than 2000 episodes of in-patient discharges and deaths due to an alcohol-related disease [6].

Nonetheless, the Hong Kong Special Administrative Region (HKSAR) Government halved the duty on beer and wine with an alcoholic strength of not more than 30% in 2007 [7], and abolished it altogether in 2008 [8]. The duty rate on spirits with an alcoholic strength of more than 30% remained at 100% [8]. These unprecedented and anti-public health policies were aimed to help Hong Kong develop into an international wine trading hub, at the cost of increased alcohol-related harms to public health [9]. The Working Group on Alcohol and Health of the Department of Health noticed a surge in the alcohol consumption per capita in Hong Kong in 2008, which was attributed to the lowered price of beer and wine in the same year [4]. This echoed a meta-analysis of 112 studies, which found an inverse relation of alcohol tax or price with consumption [10]. In Finland, the one-third reduction of excise duties on alcoholic beverages in 2004 resulted in a clear rise in alcohol consumption and related harms [11], including hospitalization rate [12] and number of sudden deaths [13]. The drastic change of beer and wine tax policy in Hong Kong presents a unique opportunity to study alcohol drinking using infodemiology.

### Infodemiology and Infoveillance

Infodemiology is a portmanteau of information and epidemiology, which is, according to Eysenbach, “the science of distribution and determinants of information in an electronic medium, specifically the Internet, or in a population, with the ultimate aim to inform public health and public policy” [14,15]. It is based on a bidirectional relation between population health status/attitudes/behavior and information patterns on the Internet [15,16]. Originally used to identify inaccurate health information on the Internet [17], it was later found that search engine query data could predict influenza epidemics [18]. Given its implication in public health and policy, infodemiology has since been used as a complement to traditional epidemiological studies [15,16], using analytical methods and metrics such as keyword prevalence and prevalence ratio [14]. The longitudinal tracking of infodemiology metrics for surveillance and trend analysis is called infoveillance [14,15].

Over the past decade, efforts have been made to overcome the difficulties in aggregating and analyzing the vast, unstructured information from the online database [15,16]. Examples of infodemiology within the public health sector include detection of disease outbreak or incidence by tracking search queries [16,18-20], investigating online search behavior for suicide-related information [21], monitoring public reactions towards health-related policy and campaigns [22,23], and identifying public health concerns from user posts in social networking sites such as Twitter [24]. Several computer tools have also been developed that allow more effective infodemiological analysis (eg, Technosocial Predictive Analytics [25], Infovigil [15], Global Public Health Intelligence Network, and HealthMap [26]). All these are underpinned by the technology of Web 2.0, which features individuation, open property, sociality, and microcontent [27]. One established example of Web 2.0 is online social networking.

### Online Social Networking, Blogs, and Blog Searching

Online social networking is constantly evolving. With proper mining and analytic technique [28], it might be possible to extract useful information from these networking sites for research purpose. Blogs (also called weblogs) are a kind of social networking website that is regarded as a relatively new form of mainstream personal communication [29,30]. They are characterized as being personalized, Web-based, community-supported, and automated [31]. Blog contents are versatile. They could be about the blogger’s life, commentaries, ideas, and emotions. They are also used to form and maintain community forums [32]. While some blogs are employed for political, educational, and commercial purposes [33], most are likened to diaries and are referred to as personal blogs [34]. People using blog services are referred to as bloggers.

Like other social networking sites, blogs are featured by their time stamps, consumer-generated content, and expansile database, making them potentially useful for longitudinal data retrieval and analysis [35,36]. In fact, blog analysis has become increasingly popular in various domains. Examples of its application include assessing a company’s image strength or customer product, monitoring public opinion in presidential elections, evaluating public reaction to disasters, tracing online hate groups or people with suicidal intent, studying youth cultures, and analyzing linguistic patterns [34,37-40]. The prerequisite of blog analysis for public surveillance is that a large proportion of the population should use the social Web services regularly, so that online information can be kept up-to-date and truly reflect the contemporary interest or concern of the community [25].

### Internet Use and Blogging in Hong Kong: Applying Blog Searches in Local Public Health Research

Internet use is ubiquitous in Hong Kong. In a survey by the HKSAR Government, Internet penetration of local population continued to rise over the years, from 56.9% in 2005 to 72.8% in 2012. Almost 70% of the online population were adolescents and adults aged 10-44 [41]. They also constituted the largest population engaging in online social networking activities including blogs and forums [42]. This was consistent with an older study by Blog-You.com and IN-Media in 2005, which found over 90% of local bloggers were aged 16-35 [43]. Infodemiology using blog searching data is therefore useful for studying important health issues among Hong Kong adolescents and adults, such as alcohol use.

There are different methods of blog analysis. Some (eg, time series scanning, semantic analysis) require special software and significant investment of time and computing resources [35]. This has greatly reduced its practicability in public health research done by clinicians who might not otherwise know much about computer programming. On the other hand, blog searching using blog search engines, which are freely available online, provides a technically easy, straightforward, and user-friendly method to extract data from blogs. Unlike Web search engines, blog search engines mainly index blog posts and are dedicated to searching information from blog posts only, ignoring the rest of the database [44]. Since each blog post is time-stamped, a blog search engine could search for date-specific blog posts, allowing longitudinal blog tracking, even retrospectively.

Local public health research using blog search and Chinese keywords is rarely done at the moment. Should blog searching data be correlated with local epidemiological data, clinicians (and policy makers) could readily replace traditional surveillance methods with blog searching—which is real-time, cheap, and fast—for public health tracking. Even if there is no correlation, an explanatory study like this would still contribute to the development of health informatics by demonstrating the challenges of Chinese blog searches in an otherwise English-dominated research environment. The clear rise of alcohol consumption in Hong Kong due to zero beer and wine tax provides a sound framework under which blog searching data can be validated against local epidemiology.

### Study Objectives and Hypotheses

Using currently available search tools and Web resources, this study aimed to: (1) assess and explain the online use of alcohol-related Chinese keywords, and (2) validate blog searching as an infoveillance method to survey changes in the drinking patterns (eg, alcohol type) of Hong Kong people (represented by bloggers in a Hong Kong–based Chinese blog service provider) following changes to the beer and wine tax in 2007-8.

Following are the hypotheses of this study:

(H1) The online popularity of alcohol-related concepts, in particular “beer or wine”, increased among Chinese bloggers after 2007-8 when local tax policy on beer or wine changed.

(H2) Infodemiological data (represented by prevalence rate and ratio of alcohol-related concepts) correlated significantly with local epidemiological data (represented by per capita alcohol consumption).

## Methods

### Study Design

To the best of our knowledge, this was the first research done in Hong Kong using blog searches to study a public health–related topic. The issue of alcohol drinking was chosen because of its public health interest and clear impacts by tax policy changes. Blogs were targeted for data extraction because regional interest could be maximized by choosing a blog service provider with the highest local rank and visitors, unlike other social networking sites such as Twitter or Facebook, which tend to cover a wide geographical area.

There were two main sets of data in this study: (1) infodemiology and (2) epidemiology. Infodemiological data stemmed from existing blogs indexed by a specific search engine, whereas epidemiological data were obtained from government documents covering the issues of public health. To reduce expertise and technology investment, this study used a free Web-based blog search engine, Google Blog Search, to extract data from the archives of a Hong Kong–based blog service provider, MySinaBlog, from 2005-2010. Three groups of Chinese keywords were used, each representing a specific alcohol-related concept. They were (1) “alcohol” (ie, the control concept), (2) “beer or wine”, and (3) “spirit”. The resulting blog posts were analyzed quantitatively using infodemiological metrics and correlation coefficients, and qualitatively by manual effort. The infodemiological metrics were (1) apparent prevalence, (2) actual prevalence, (3) prevalence rate, and (4) prevalence ratio. Pearson and Spearman correlations were calculated for prevalence rates and ratios with respect to per capita alcohol consumption in the same years. Manual analysis included (1) blog author characteristics (ie, authorship, sex, and age), and (2) blog content (ie, frequency of keywords, description of a discrete episode of alcohol drinking, drinking amount, and genres). The prevalence rate and ratio were used to assess the online popularity of alcohol-related concepts, whereas the correlation analysis and manual analysis were used to validate blog searching data as an infoveillance method for population survey.

### Collection of Infodemiological Data

#### Blog Service Provider

The online database of the blog service provider enabled search tasks and collection of infodemiological data. Inclusion criteria were (1) free of charge, (2) currently under service, and (3) last updated in or after 2010. As such, 19 blogging sites were enlisted from 852.com [45], which was a Hong Kong-based Web directory, and TopTenREVIEWS [46], a media review website. Their online traffic data were obtained from two Web information companies, Alexa Internet [47] and StatsCrop [48], shown in Table 1. They were excluded if (1) the only available figures were from their non-blog domain server, or (2) data were not available. The rest were compared in terms of their local popularity (measured by Alexa traffic rank in Hong Kong and percentage of daily visitors from Hong Kong) and primary country/server location to maximize the regional interest. MySinaBlog, a Hong Kong-based blog service provider with a local rank of 201 and more than half of its visitors from Hong Kong, was eventually selected.

**Table 1:** Alexa traffic rank in Hong Kong, percentage of daily visitors from Hong Kong, and primary country^a^ of selected free blogging sites (as on April 12, 2013).

URL	Alexa traffic rank in Hong Kong	Percentage of daily visitors from Hong Kong	Primary country
blogcity.me	1145	74.2	Hong Kong
blog.mingpao.com	94^b^	56.8^b^	Hong Kong
blog.yahoo.com/explorer/hk	4^b^	0.8^b^	United States
hk.xanga.com	571	5.4	United States
lifestream.aol.com	Data not available	Data not available	United States
mysinablog.com	201	50.8	Hong Kong
qooza.hk	417	35.7	Hong Kong
showhappy.net	Data not available	Data not available	United States
spaces.live.com	Data not available	Data not available	Iran
space.gogo.la	1352^b^	67.0^b^	Hong Kong
space.uwants.com/html/blog.html	22^b^	57.4^b^	Hong Kong
wordpress.com	Data not available	Data not available	United States
www.blogger.com	Data not available	Data not available	India
www.ezhk.net	Data not available	Data not available	Hong Kong
www.hkflash.com/diary	7012^b^	25.6^b^	South Korea
www.livejournal.com	Data not available	Data not available	Russia
www.mocasting.com	16,435	51.8	China
www.myspace.com	Data not available	Data not available	United States
www6.mobichai.com/blog	Data not available	Data not available	Hong Kong

^a^Or server location if the primary country was not known.

^b^Representing the only available data from its non-blog domain server.

#### Blog Search Engine and Search Query

The capabilities and limitations of 11 blog search engines were compared in one study by Thelwall [44]. Among them, Google Blog Search was the only one equipped with all of the following features: (1) full Boolean search, (2) user-specified date or date range search, (3) URL search, (4) language selection, and (5) word location. It was therefore employed in the present study.

In each search query, the following were included: (1) alcohol-related Chinese keywords connected by the Boolean operator “OR”, and (2) URL of the blog service provider expressed as “site:mysinablog.com”. To obtain the total number of blog posts, the keywords were substituted by a space. The date was specified as January 1 to December 31 of each year from 2005-2010. The timeframe was so decided because MySinaBlog started to run their service in 2005 [49], and epidemiological data regarding alcohol consumption per capita in Hong Kong was only up to 2010 [4]. The search results (ie, number of matched blog posts) were taken for infodemiology analysis.

#### Alcohol-Related Concepts and Keywords

##### Overview

Specific groups of alcohol-related keywords formed the basis of blog searching in this study. Each group corresponded to a concept and comprised multiple keywords connected by the Boolean operator “OR” (which would return blog posts containing any of the search terms) to explore the same concept, as suggested by Eysenbach in his framework on infodemiology and infoveillance [14]. The concepts were (1) “alcohol”, (2) “beer or wine”, and (3) “spirit”. They were chosen because beer and wine contained an alcohol strength of not more than 30%, and it was upon this group of liquors that the HKSAR Government halved the duty in 2007 and then abolished it in 2008. To better compare the concepts of “beer or wine” and “spirit”, “alcohol” was chosen as the control (ie, the broader) concept to calculate the prevalence ratios, in addition to the prevalence rates.

**Figure 1:**
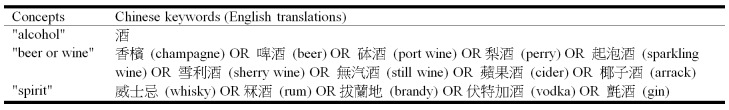
Alcohol-related concepts and their corresponding Chinese keywords typed into the search field.

Figure 1 shows the concepts and keywords that were typed into the search field. All keywords were in traditional Chinese, which was more often used than simplified Chinese or English in Hong Kong. Compared to using keywords in both Chinese and English, it could (1) enhance the homogeneity of the output data, and (2) reduce the size of the output data to ease subsequent manual analysis.

##### Keyword “Alcohol”

The English word “alcohol” was translated to Chinese using Lin Yutang’s Chinese-English Dictionary of Modern Usage (Online Version) [50]. In general, two or more Chinese characters made up a Chinese word. Different Chinese words might share the same meaning, whereas some Chinese words might have more than one meanings. To reduce confusion and widen the search coverage, only the Chinese character shown in Figure 1 was used for alcohol, instead of other Chinese words with the same meaning. It should be noted, however, that some Chinese translations were simply taken from the phonics in English without including the Chinese character for alcohol (eg, champagne, whisky, brandy). It would be impossible (and impractical) to guarantee a full coverage of the search results for all kinds of beer, wine, and spirit using the single Chinese character for alcohol shown in Figure 1. Nonetheless, it already provided the largest inclusion as a control concept of this study.

##### Keywords “Beer or Wine” and “Spirit”

Keywords that belonged to the concepts “beer or wine” and “spirit” were chosen from a document released by the Customs and Excise Department of the HKSAR Government [51], which related to the budget proposals about changes in the beer and wine tax. They contrasted the impacts brought about by the tax policy. Generic terms with variable alcoholic strength were excluded (eg, sake, sugar spirit, reprocessing Chinese liquor). The rest were categorized under “beer or wine” if the alcohol strength was not more than 30%, or “spirit” if otherwise.

### Collection of Epidemiological Data

Table 2 shows the per capita alcohol consumption extracted from a report released by the Department of Health of the HKSAR Government in 2011 [4]. It was adopted in our study because it was (1) freely accessible, (2) presented in a longitudinal form, and (3) subgrouped according to alcohol types. Data from 2011 were not available, and no updates of the data were seen hitherto.

### Quantitative Analysis

#### Infodemiological Metrics: Apparent Prevalence, Actual Prevalence, Prevalence Rate, and Prevalence Ratio

**Figure 2:**
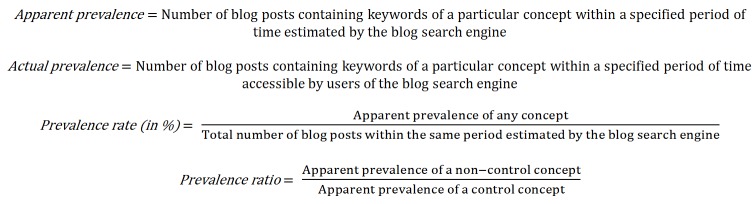
Definitions/formulae of the infodemiological metrics.

Eysenbach advocated the use of relative indicators such as rates and ratios in lieu of absolute figures to represent information prevalence since the number of websites was constantly changing [15]. With slight modifications of his proposal, the following infodemiological metrics were used to indicate the online popularity of the concepts in blog posts: (1) apparent prevalence, (2) actual prevalence, (3) prevalence rate, and (4) prevalence ratio. The definitions/formulae of the metrics are shown in Figure 2. The apparent prevalence referred to an estimate by the blog search engine, and the actual prevalence was confirmed by the researcher who did the counting while accessing each website. The apparent prevalence instead of actual prevalence was used to calculate the prevalence rate because the total number of blog posts was again an estimate by the blog search engine. Similarly, the prevalence ratio was calculated using apparent prevalence instead of actual prevalence to avoid confusion in the correlation analysis.

**Table 2:** Total and per capita alcohol consumption in Hong Kong from 2005-2010 (adapted from the Department of Health of the HKSAR Government).

Year	Total pure alcohol consumption (in liters)		Population aged ≥15 years	Per capita alcohol consumption (in liters)	
	Beer and wine	Spirit		Beer and wine	Spirit
2005	9,382,633	5,376,813	5,844,300	1.61	0.92
2006	9,442,114	5,586,247	5,918,000	1.60	0.94
2007	9,878,382	5,927,246	6,004,700	1.65	0.99
2008	12,309,905	5,946,634	6,075,400	2.03	0.98
2009	11,973,446	4,244,254	6,130,300	1.95	0.69
2010	11,252,645	5,156,867	6,209,800	1.81	0.83

#### Pearson and Spearman Correlations

A correlation analysis was done to validate the use of infodemiological data in surveying the drinking patterns of the local population, as shown in Table 3. Essentially, the infodemiological data (ie, prevalence rates and ratios) acted as the independent variable whereas the epidemiological data (ie, per capita alcohol consumption) acted as the dependent variable. Pearson and Spearman correlations were calculated using the Statistical Package for the Social Sciences (SPSS).

### Qualitative Analysis

Once blog searching was done, the blog posts were saved in .html files for subsequent analysis to avoid discrepancy due to time lag. The manual analysis focused on (1) blog author characteristics (ie, authorship, sex, and age), and (2) blog content (ie, frequency of keywords, description of a discrete episode of alcohol drinking, drinking amount, and genres). They would provide further information about the validity of utilizing blog searching data in an epidemiological survey. Their subcategories and criteria are listed in Table 4.

Blog posts with “alcohol” keywords were not included for manual analysis since a large portion of them were expected to overlap with those that contained “beer or wine” and “spirit” keywords. They might not be particularly helpful in analyzing the drinking pattern (eg, choice of alcohol) of the population.

It was noteworthy that most of the free text analytic tools did not support Chinese language and had no way to identify position of the keywords within a blog (eg, header, main body, sidebar, footer, and comment). Currently available concordancers for Chinese language were not too user-friendly as they lacked an external encoder/decoder, keyword-in-context (KWIC) format, or built-in dictionaries for semantic analysis or opinion mining [52]. This was why manual analysis was chosen in this study as a preliminary measure to explore the blog author characteristics and blog content.

**Table 3:** Correlation of infodemiological and epidemiological data.

Infodemiological data	Epidemiological data
“alcohol” prevalence rate	Per capita consumption of all alcoholic types
“beer or wine” prevalence rate	Per capita consumption of beer and wine
“spirit” prevalence rate	Per capita consumption of spirits
“beer or wine” / “alcohol” prevalence ratio	Per capita consumption of beer and wine
“spirit” / “alcohol” prevalence ratio	Per capita consumption of spirits

**Table 4:** Categories, subcategories, and criteria for manual analysis of blog posts containing “beer or wine” and “spirit” keywords in MySinaBlog from 2005-2010.

Categories	Subcategories
Authorship	(1) Single author, or (2) multiple authors
Sex	(1) Female, (2) male, or (3) unknown
Age	(1) Below 18 years old, (2) 18 years old and above, or (3) unknown
Frequency of keywords	Not applicable
Description of a discrete episode of alcohol drinking	(1) Yes, or (2) no
Drinking amount	(1) Binge drinking, (2) non-binge drinking, and (3) undetermined
Genres	(1) Name of a place/person/entity not belonging to alcohol, eg, lyrics, (2) recipe/dish name, (3) news/copied article from an external source, (4) story narrative/film synopsis, (5) health/educational information, (6) non-opinionated featured article, (7) personal diary/opinion/emotional outlet, or (8) more than one of the above

## Results

### Overview

The blog search was done on April 12, 2013, and the manual analysis was completed by researcher, KL Chan, in the subsequent week. The results are described below.

### Quantitative and Correlation Analysis

#### Apparent and Actual Prevalence

Table 5 shows that the total number of blog posts in MySinaBlog increased dramatically within 5 years’ time, from less than 500 in 2005 to more than 20,000 in 2010. An increasing trend was also observed for the apparent prevalence of “alcohol”, “beer or wine”, and “spirit” except in 2010 when that of “beer or wine” and “spirit” dropped compared to the year before.

The apparent prevalence of “alcohol” was consistently higher than that of “beer or wine” and “spirit”, which made sense as “alcohol” was the control concept. However, in 2005 the apparent prevalence of “alcohol” was only 3, compared to that of “beer or wine”, which was 5. This might be explained by translation difficulties where the Chinese character of “alcohol” did not cover all keywords of “beer or wine” and “spirit”. On the other hand, the apparent prevalence of “beer or wine” was higher than that of “spirits” in 2005-2007 and 2010. In 2008 however, the two were equal, and in 2009, the apparent prevalence of “spirit” surpassed that of “beer or wine” by a difference of 17.

The discrepancies between apparent and actual prevalence became more obvious when their values enlarged in all three concepts. For example, the apparent prevalence of “alcohol” in 2005 was 3 and was the same as the actual prevalence; but in 2006, as the former increased to 26, the two differed by 12. By the time the apparent prevalence of “alcohol” reached up to 1390 in 2010, the actual prevalence of “alcohol” was only 195, representing a difference of 1195. Of particular note, the actual prevalence of “spirits” of 12 in 2008 and 13 in 2009 was much lower than its apparent prevalence of 73 and 115, respectively, due to spams in blogs. The trends of the apparent and actual prevalence were grossly symmetrical for “alcohol” and “beer or wine” except that in 2010, the actual prevalence of “beer or wine” peaked instead of waning.

**Table 5:** Total number of blog post, apparent and actual prevalence of alcohol-related concepts in MySinaBlog from 2005-2010.

Year	Total number of blog posts	Apparent prevalence (actual prevalence)		
		“Alcohol”	“Beer or wine”	“Spirit”
2005	394	3 (3)	5 (5)	0 (0)
2006	1810	26 (14)	16 (15)	3 (3)
2007	5620	120 (59)	27 (15)	5 (5)
2008	11,500	1180 (150^a^)	73 (28)	73 (12^a,b^)
2009	16,000	1290 (190^a^)	98 (25)	115 (13^a,b^)
2010	20,400	1390 (195)	70 (41^c^)	3 (3)

^a^Final figures included those blog posts that were initially hidden and prompted by the blog search engine.

^b^After excluding blogs with spams that actually contained no keywords.

^c^After excluding one blog post that was inaccessible due to security reasons.

#### Prevalence Rate and Correlation Coefficients

Table 6 shows that the prevalence rate of “alcohol” followed an inverted V shape, increasing steadily from 0.76% in 2005 to 2.14% in 2007, peaking at 10.26% in 2008, and decreasing to 8.06% in 2009 and then to 6.81% in 2010. The prevalence rate of “beer or wine” declined over the years with its first trough of 0.48% in 2007 and second trough of 0.34% in 2010. The prevalence rate of “spirit” was quite the opposite, initially hovering at a low level of 0% to 0.17% in 2005-2007, then surging up to 0.72% in 2008-2009, and eventually falling back to 0.01% in 2010.

The prevalence rate of “alcohol” was consistently higher than that of “beer or wine” and “spirit” except in 2005 when the prevalence rate of “alcohol” was only 0.76%, compared to that of “beer or wine”, which was 1.27%. This might be explained by translation difficulties as stated before. The prevalence rate of “spirit” was the lowest among all three concepts in 2005-2007 and 2010. However, in 2008, it tied with the prevalence rate of “beer or wine”, and in 2009, exceeded it altogether.

Per capita consumption of alcohol correlated strongly with the prevalence rate of “alcohol” (Pearson correlation=0.81, *P*=.05; Spearman correlation=1.00, *P*<.001). The linear relationship was marginally significant and the nonlinear relationship was significant. The prevalence rate of “beer or wine” was negatively and moderately correlated with per capita consumption of beer and wine (Pearson correlation=-0.48, *P*=.34; Spearman correlation=-0.43, *P*=.40). Both were nonsignificant. Similarly, the prevalence rate of “spirit” had a moderate negative linear correlation (Pearson correlation=-0.40, *P*=.43) and a weak negative nonlinear correlation (Spearman correlation=-0.09, *P*=.87) with per capita consumption of spirits. Again, both were nonsignificant.

#### Prevalence Ratio and Correlation Coefficients

Table 7 shows that the prevalence ratio of “beer or wine” / “alcohol” declined as a whole, troughing at 0.06 in 2008 and 0.05 in 2010. The prevalence ratio of “spirit” / “alcohol”, on the other hand, peaked at 0.12 in 2006 and 0.09 in 2009. The former was higher in 2005-2007 and 2010. However, in 2008, the two tied, and in 2009, the prevalence ratio of “spirit” / “alcohol” reached 0.09, surpassing that of 0.08 for “beer or wine” / “alcohol”.

The prevalence ratio of “beer or wine” / “alcohol” had a strong negative correlation with per capita consumption of beer and wine (Pearson correlation=-0.65, *P*=.16; Spearman correlation=-0.77, *P*=.07). The correlation coefficients were nonsignificant. The prevalence ratio of “spirit” / “alcohol” was also negatively correlated with per capita consumption of spirits but only weakly (Pearson correlation=-0.10, *P*=.85; Spearman correlation=-0.03, *P*=.96). Again, both correlation coefficients were nonsignificant.

**Table 6:** Prevalence rate of alcohol-related concepts in MySinaBlog and correlation coefficients compared with per capita consumption of the same alcohol types in Hong Kong from 2005-2010.

		Prevalence rate (%)		
		“Alcohol”	“Beer or wine”	“Spirit”
**Year**				
	2005	0.76	1.27	0
	2006	1.44	0.88	0.17
				
	2007	2.14	0.48	0.09
	2008	10.26	0.63	0.63
	2009	8.06	0.61	0.72
				
	2010	6.81	0.34	0.01
**Correlation coefficients (*P value*)**				
	Pearson	0.81 (.05)	-0.48 (.34)	-0.40 (.43)
	Spearman	1.00 (<.001)	-0.43 (.40)	-0.09 (.87)

**Table 7:** Prevalence ratios of alcohol-related concepts in MySinaBlog and correlation coefficients compared with per capita consumption of the same alcohol types in Hong Kong from 2005-2010.

		Prevalence ratio (%)	
		“Beer or wine” / “alcohol”	“Spirit” / “alcohol”
**Year**			
	2005	1.67	0
	2006	0.62	0.12
	2007	0.23	0.04
	2008	0.06	0.06
	2009	0.08	0.09
	2010	0.05	0.00
**Correlation coefficients (*P value*)**			
	Pearson	-0.65 (.16)	-0.10 (.85)
	Spearman	-0.77 (.07)	-0.03 (.96)

### Qualitative Analysis

#### Blog Author Characteristics

**Figure 3:**
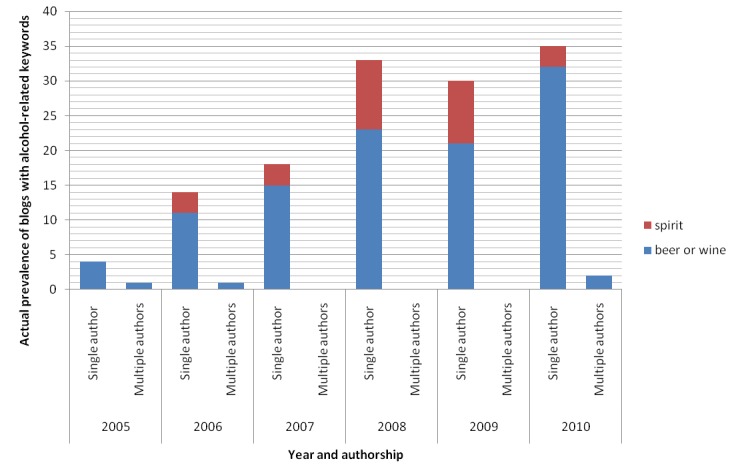
Actual prevalence of blogs with alcohol-related keywords in MySinaBlog from 2005-2010 classified according to authorship (corporational or organizational blogs were counted as multiple authors; different blog posts by the same registered user in the same year were counted as one blog).

**Figure 4:**
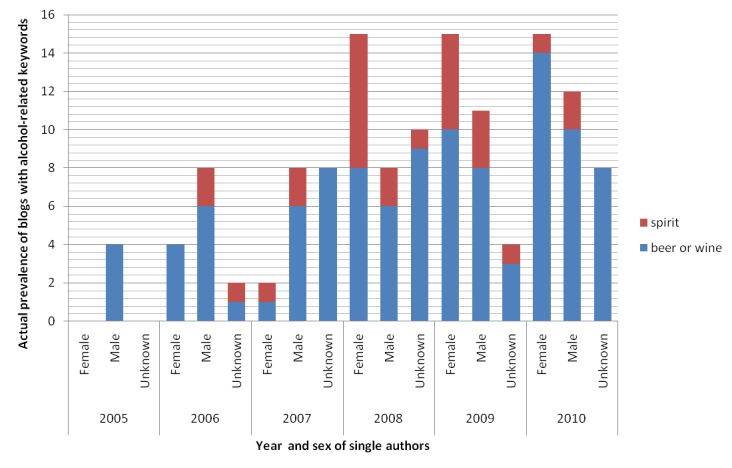
Actual prevalence of blogs with alcohol-related keywords in MySinaBlog from 2005-2010 classified according to sex of the single authors.

**Figure 5:**
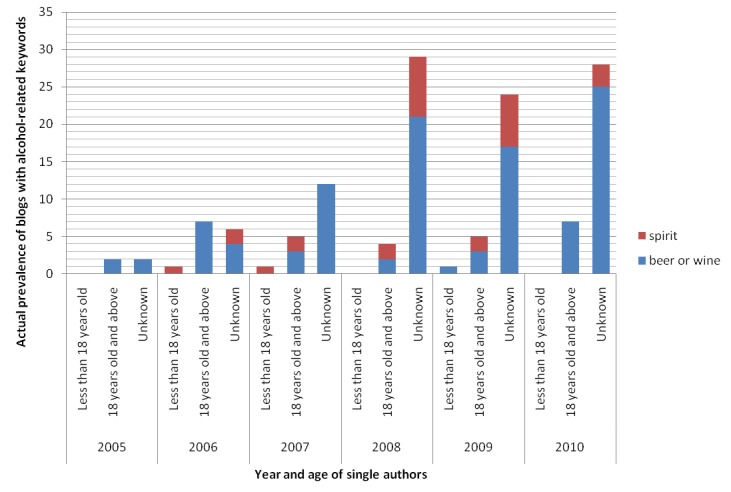
Actual prevalence of blogs with alcohol-related keywords in MySinaBlog from 2005-2010 classified according to age of the single authors.

Figure 3 illustrates that a substantial number of blogs with alcohol-related keywords in MySinaBlog from 2005-2010 were written by single authors (97.1%, 134/138). For those single authors whose sex identity was known, their sex distribution was equal (female=38.1%, 51/134; male=38.1%, 51/134; unknown=23.9%, 32/134) (Figure 4). Most single authors also did not indicate their age (unknown age=75.4%, 101/134), while the rest were mostly adults (18 years old or above=22.4%, 30/134) (Figure 5). All parameters appeared to increase with time, possibly explained by the increase in the total number of blogs.

#### Blog Content

**Figure 6:**
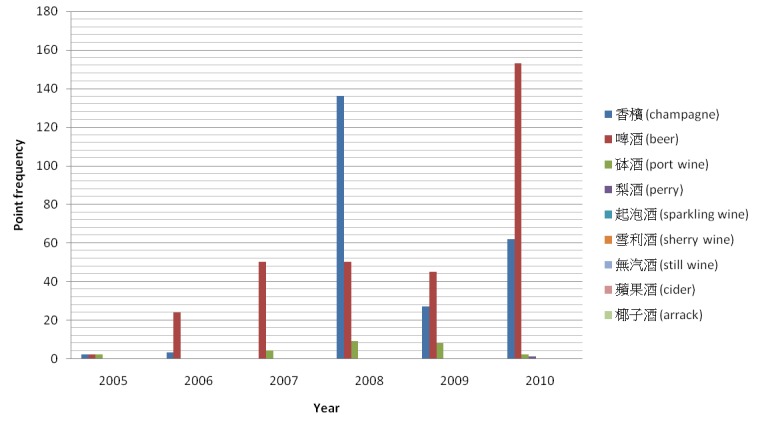
Point frequency of "beer or wine" keywords in main body of the blog posts of MySinaBlog from 2005-2010.

**Figure 7:**
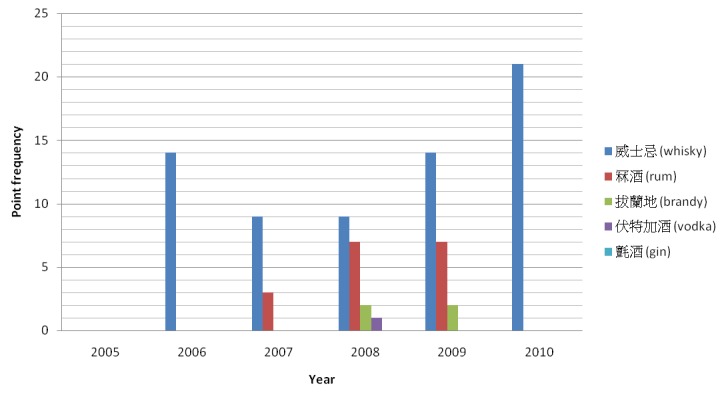
Point frequency of "spirit" keywords in main body of the blog posts of MySinaBlog from 2005-2010.

As shown in Figures 6 and 7, not all alcohol-related keywords were found in the blog posts of MySinaBlog in 2005-2010. Among “beer or wine” keywords, “beer” was the most common. It had an accumulated frequency of 324 from 2005-2010 and peaked at a point frequency of 153 in 2010. “Champagne” was the second most common, followed by “port wine”, and lastly “perry”. As for “spirit” keywords, “whisky” was the most common. It had an accumulated frequency of 67 from 2005-2010 and peaked at a point frequency of 21 in 2010. “Rum” was the second most common, followed by “brandy”, and lastly “vodka”. The point frequency of the keywords seemed to rise over the years, possibly explained by an increase in the actual prevalence of the blog posts.

**Figure 8:**
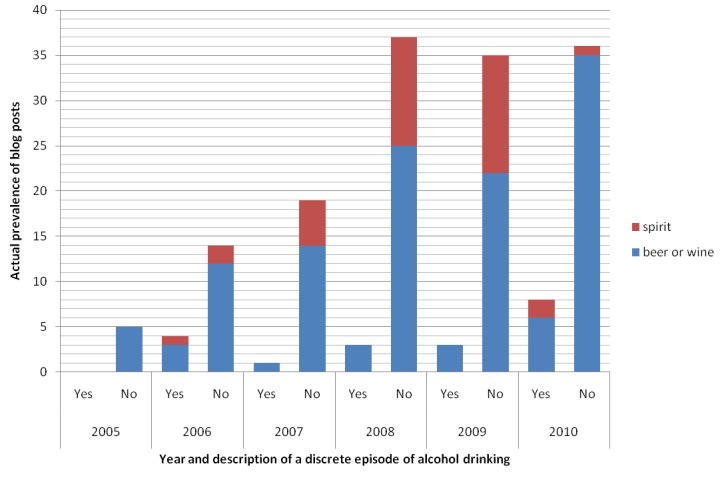
Actual prevalence of blog posts with alcohol-related keywords in MySinaBlog from 2005-2010 classified according to description of a discrete episode of alcohol drinking (alcohol used for cooking was excluded).

**Figure 9:**
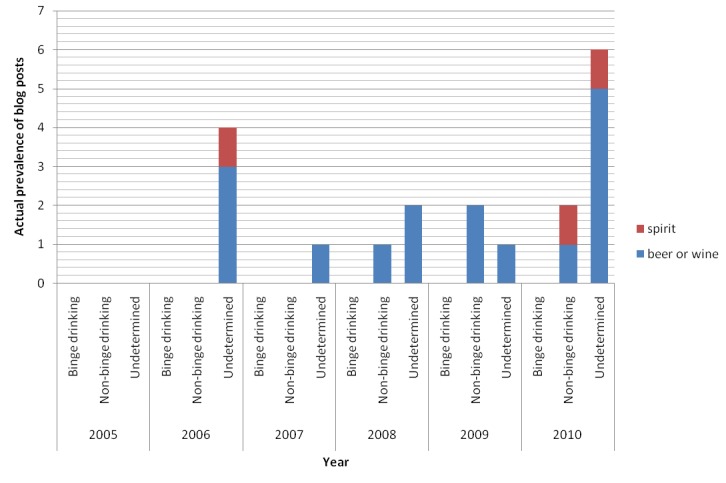
Actual prevalence of blog posts with alcohol-related keywords and description of a discrete episode of alcohol drinking in MySinaBlog from 2005-2010 classified according to drinking pattern (binge drinking defined as 5 alcoholic drinks in a row within a couple of hours).

**Figure 10:**
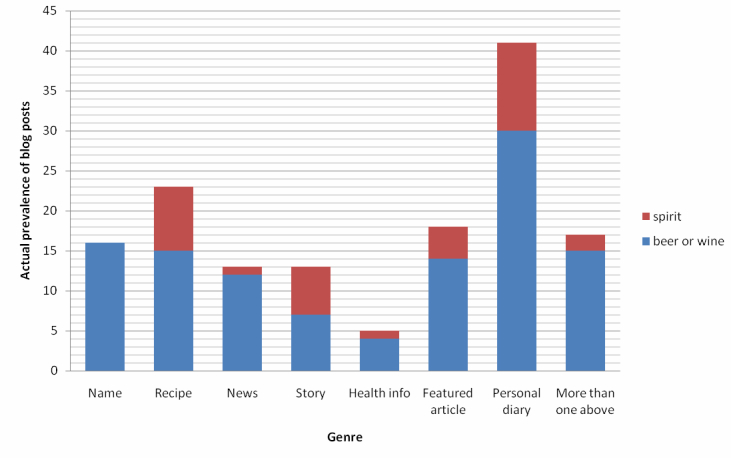
Actual prevalence of blog posts with alcohol-related keywords but no description of a discrete episode of alcohol drinking in MySinaBlog from 2005-2010 classified according to genre.

As shown in Figure 8, not all blog posts actually described a discrete episode of alcohol drinking (alcohol type specified by the keyword) by the author in Hong Kong and in the same year when the blog post was published. In fact, only 11.5% (19/165) of them did so, with limited information regarding the drinking amount and duration. It was thus difficult to differentiate between binge and non-binge drinking (binge drinking=0%; non-binge drinking=26.3%, 5/19; undetermined=73.7%, 14/19) (Figure 9; [35]). The others were mostly personal diary, opinion, or emotional outlet (28.1%, 41/146) (Figure 10). In Figure 10, the name is the name of a place, person, or entity not belonging to alcohol, eg, lyrics; recipe is recipe or dish name; news is the news/copied article from an external source; story is the story narrative/film synopsis; health info is the health or educational information; featured article is the non-opinionated featured article; personal diary is a personal diary, opinion, or emotional outlet. The immediate text surrounding the keyword(s) was first examined. If a decision was not made or the keywords were too disperse, the entire blog post was examined. Former options should be considered before latter ones.

## Discussion

### Changes in the Online Popularity of Alcohol-Related Concepts

The online popularity of alcohol-related concepts was best represented by their prevalence rate and ratio, which normalized the effect of any changes in the total number of blogs [14]. In general, the concept “alcohol” was most popular in 2008. The concept “spirit” also experienced a short-lasting and somewhat erratic rise in its online popularity in 2008-2009. The concept “beer or wine”, in contrast, became increasingly unwelcome with an overall declining trend in its online popularity over the years. The hypothesis that alcohol-related concepts became more popular after 2007-2008 was true only for “alcohol” and “spirit” but not “beer or wine”.

One possible reason for the increase in the online popularity of “spirit” keywords in 2008-2009 was the presence of spam in the same years. This was masked in the apparent prevalence, which was used to calculate the prevalence rate and ratio. Indeed, after excluding the data in 2008-2009, the prevalence rate and ratio of the concept “spirit” remained relatively stable at a low level. The false elevation in the online popularity of “spirit” might also explain the peak prevalence rate of “alcohol” in 2008, although the latter appeared much larger in amplitude and there was still a chance for a genuine rise in keywords which were not included in “beer or wine” or “spirit” but “alcohol”. The downhill course in the online popularity of “beer or wine” was probably a true reflection of bloggers’ decreased interest over the topic. However, its relation to local drinking pattern remained doubtful, since many of the blog posts were in fact not describing a discrete episode of alcohol drinking. The same argument held true for the concepts “spirit” and “alcohol”.

### Validating Blog Searching as an Infoveillance Method for Surveying Drinking Patterns

Validation of blog searching data depended on correlation analysis and manual analysis of blog author characteristics and contents. The prevalence rate of “alcohol” was the only parameter that had a significant nonlinear and a marginally significant linear correlation with per capita alcohol consumption. The other correlations were all nonsignificant, although many of them demonstrated moderate to strong strengths. The hypothesis that infodemiological data correlated significantly with local epidemiological data was true only for “alcohol” prevalence rate. The statistical nonsignificance of other infodemiological metrics might be explained by the small number of blog posts relative to the population. This in turn could be attributed to the following:

1. Choice of keywords. The list of keywords for “beer or wine” and “spirit” could never be exhaustive since their types were many and expressions by bloggers were highly variable. Mixed code of Chinese characters and English letters was not uncommon for online communications among Hong Kong people. Some of them would actually type Cantonese (dialect of Yue Chinese) rather than standard Chinese [53]. They might use a different Chinese word as the translation of the same liquor. They might also use the brand name of the liquor they took. This was partly reflected by the frequency of individual alcohol-related keyword in the blog posts, showing that some were not used by bloggers at all. All these added difficulties in selecting the appropriate keywords that gave an adequate coverage within the word limit of the search query.
2. Passive blogging behavior among Hong Kong people. In a survey done by the HKSAR Government in February to April 2011, 53.4% of Internet users had browsed contents at forums or blogs in the preceding 12 months, yet only around 15.8% had compiled or created webpages or blogs in the same period [42].

From the manual analysis, most blogs were managed by single authors, meaning that the number of blogs could be used to represent the number of individual attendants in a population survey. The sex distribution of the single blog authors was close to the local population, but their age range was slightly inclined to 18 years old and above [54]. It should be noted, however, that many of the bloggers did not disclose their identity online, making validation difficult.

Many of the blog posts were not about a discrete episode of alcohol drinking but personal diary, opinion, or emotion outlet. This was not surprising as new genres of blogs continued to emerge [55]. Rather than just being informative, many blogs were affective in nature requiring semantic analysis for meaningful interpretation [25]. While it was unlikely that bloggers recorded their alcohol intake on each occasion, they might reveal their understanding on alcohol drinking when commenting on a particular event, answering a particular question, and describing a childhood incident, etc, hence, the presence of alcohol-related keywords. While there were inadequate clues to support that changes in the online popularity of the alcohol-related keywords were related to an altered drinking pattern of the local population, one should not ignore its social implications and disregard its role in evaluating public reactions towards health-related policy including the zero beer and wine tax.

### Research Limitations and Solutions

Using blogs as the source of information had several inherent limitations. For example, demographic data of individual blogger such as gender, age, and race might be deficient or disguised; bloggers tended to share common interests and backgrounds that were probably different from those of the general population; and acquisition of precise data such as drinking patterns was often difficult. In order to construct a larger framework in a timely and efficient manner, informatics researchers often had to compromise the individuality of each blogger by using certain infodemiological metrics. Moreover, language usage by bloggers tended to be complex and not easily decoded by the frequency of some pre-determined keywords. As a solution, semantic analysis of individual blog post might be useful to explore bloggers’ opinions towards drinking, provided enough technical support. Face-to-face interviews and questionnaires might be conducted with individual bloggers to elaborate their viewpoints, preferably those who appeared to have the largest influence within a specific blog circle (using a social network analytic tool).

No single blog search engine indexed all blogs [56]. Despite its automaticity, a search engine might be subject to editorial choice and hence bias [57]. There were concerns that even in the same search engine, the search results may be different over time [14,44]. In our case, it might be explained by the (1) inherent limitation in the search algorithms of Google, which gave only an approximate estimate for query with large results, and (2) inconsistency of the search database due to a variable number of splogs (or spam blogs) and blogs that were previously not linked [35,44,56]. Of note, a large part of the Google Search algorithm was unknown to the public, aggravating sampling uncertainty in our study.

One challenge with the use of Chinese language in blog searching was that it tended to have a wide range of expressions owing to geographical difference and translation from English. Also, only a limited number of blog analytic tools supported the Chinese language. A self-designed research program with well-informed blog search algorithms and analytic functions especially for Chinese blogs would be most desirable, which would depend heavily on the availability of expertise and resources.

### Conclusion and Recommendations for Future Research

Using blog searching data from a Hong Kong–based Chinese blog service provider, we concluded the following: (1) the online popularity of alcohol-related Chinese keywords was attributed to many different factors including spam, and hence not a specific reflection of local drinking patterns, (2) correlation between infodemiological data (represented by prevalence rates and ratios of alcohol-related concepts) and epidemiological data (represented by per capita alcohol consumption) was poor, and (3) many blog posts were affective rather than informative in nature. While blog searches using pre-defined Chinese keywords might not be an ideal method to survey epidemiological data such as alcohol consumption, semantic analysis of blog content would provide invaluable information on public reactions towards health-related policy, given enough expertise and resources.

## Abbreviations

HKSAR: Hong Kong Special Administrative Region
